# *Mycobacterium avium* Subspecies *paratuberculosis* and Colorectal Cancer: Putting MAP on the Map

**DOI:** 10.3390/pathogens15060604

**Published:** 2026-06-04

**Authors:** Coad Thomas Dow

**Affiliations:** McPherson Eye Research Institute, University of Wisconsin System, Madison, WI 53701, USA; ctdow@wisc.edu

**Keywords:** *Mycobacterium avium* subsp. *paratuberculosis* (MAP), colorectal cancer, infection-associated carcinogenesis, chronic inflammation, Crohn’s disease, dysplasia, One Health, zoonosis

## Abstract

The rising incidence of early-onset colorectal cancer (CRC) remains incompletely explained despite extensive investigation into genetic, environmental and metabolic factors. Emerging evidence suggests that infectious agents may contribute to colorectal carcinogenesis. A recent study by Tehrani et al. demonstrated the presence of *Mycobacterium avium* subspecies *paratuberculosis* (MAP) in a majority of colorectal cancer lesions and in approximately half of precancerous lesions, providing a critical epidemiologic anchor. MAP, a zoonotic intracellular pathogen long associated with Crohn’s disease, exhibits biological features consistent with inflammation-driven carcinogenesis, including persistence, immune modulation and systemic dissemination. Mechanistically, MAP infection may promote tumorigenesis through chronic mucosal inflammation, epithelial barrier disruption and activation of human endogenous retroviruses (HERVs) linking persistent infection to genomic instability. Detection of MAP in early lesions argues against secondary colonization and supports a potential initiating or promoting role. Within a One Health framework, MAP represents a plausible, pleiotropic and potentially modifiable contributor to CRC. While causality remains unproven, the convergence of epidemiologic association, mechanistic plausibility and early lesion involvement warrants rigorous investigation.

## 1. Introduction

Colorectal cancer (CRC) is increasingly diagnosed in individuals under 50 years of age, with incidence rising globally despite improvements in screening among older populations [1,2,3]. Despite extensive investigation, no single unifying explanation has emerged.

The recent case–control study by Tehrani et al. provides a potentially transformative observation: *Mycobacterium avium* subspecies *paratuberculosis* (MAP) DNA is detected across the neoplastic continuum, from normal mucosa to precancerous lesions and carcinoma, with increasing prevalence along this progression [4]. The presence of MAP in early lesions is particularly notable, suggesting involvement upstream in tumorigenesis.

Infectious agents are implicated in approximately 15–20% of cancers worldwide [5]. In CRC, microbial contributors such as *Fusobacterium nucleatum*, enterotoxigenic *Bacteroides fragilis*, and genotoxic *Escherichia coli* support a model in which chronic infection drives inflammation, genomic instability, and malignant transformation [6,7,8].

Colorectal carcinogenesis itself proceeds through multiple molecular pathways, including the adenoma–carcinoma sequence, microsatellite instability (MSI) and the serrated pathway [9,10]. A shared feature across these pathways is chronic epithelial injury and genomic stress: conditions that may be initiated or amplified by persistent microbial infection. Within this framework, MAP emerges as a biologically plausible upstream contributor.

While a prior hypothesis has proposed a relationship between MAP and colorectal neoplasia, the recent demonstration of MAP across the histologic continuum from normal mucosa to precancerous lesions and carcinoma provides a substantially stronger biologic framework for considering this association. This perspective integrates these recent findings with emerging concepts in infectious carcinogenesis, chronic inflammation, microbial–host signaling and One Health biology to evaluate MAP as a candidate contributor to CRC pathogenesis.

## 2. MAP: Biology, Disease and Human Exposure

MAP is the etiologic agent of Johne’s disease, a chronic granulomatous enteritis of ruminants characterized by prolonged subclinical infection and intermittent shedding [11]. The organism exhibits marked intracellular persistence and immune evasion, enabling long-term host colonization.

Herd-level prevalence has increased substantially, with large-scale surveillance demonstrating widespread infection in dairy herds in the United States and globally [12]. MAP is shed in feces, milk, and colostrum and can persist in soil and water environments for months to years under favorable conditions [13,14]. Detection of MAP in pasteurized dairy products and water systems indicates that human exposure is common rather than exceptional [14].

These findings support the conclusion that MAP is a ubiquitous environmental organism with sustained human exposure through multiple routes.

## 3. The MAP–Human Disease Paradigm

The hypothesis linking MAP to human disease originated with the work of Chiodini et al., who identified MAP-like organisms in Crohn’s disease tissue [15]. Subsequent studies demonstrated MAP in intestinal tissue, blood, and environmental sources [16,17,18].

Naser et al. reported viable MAP cultured from the blood of patients with Crohn’s disease, providing evidence of systemic infection [16]. Later investigations using culture, phage amplification, and molecular detection confirmed these findings [19]. A 2017 international consensus concluded that MAP is a viable human pathogen detectable by multiple independent methods [20]. Importantly, Koch’s postulates have not yet been fully satisfied for MAP in Crohn’s disease or colorectal cancer, owing in part to the organism’s slow growth, intracellular persistence and diagnostic complexity. Nevertheless, accumulating molecular, microbiologic and epidemiologic findings continue to strengthen the biologic association.

Despite this body of evidence, resistance to recognizing MAP as a human pathogen persists, largely due to diagnostic challenges and entrenched autoimmune frameworks.

## 4. Diagnostic Elusiveness

**Figure 1:**
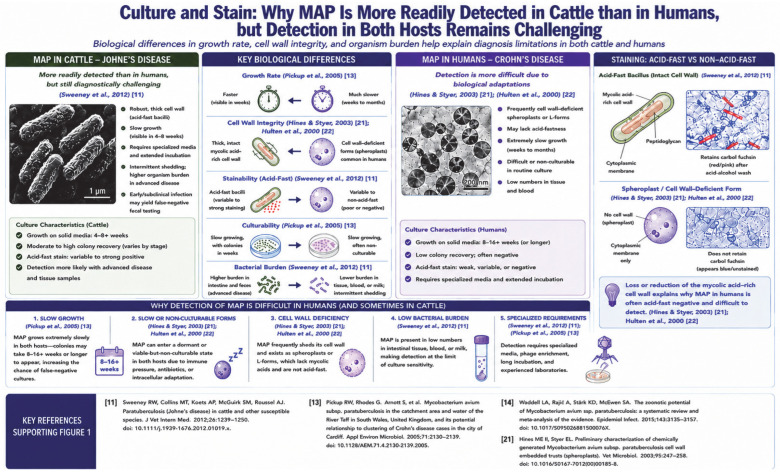
MAP is diagnostically elusive in humans due to slow growth, low tissue burden, and frequent transition to cell wall-deficient forms that stain poorly and resist routine culture. These features create a disconnect between biological presence and microbiologic detection, allowing persistent, often intracellular infection to go unrecognized. The figure highlights how this hidden persistence may sustain chronic intestinal inflammation and contribute to dysplasia and colorectal carcinogenesis. References: [11,13,14,21,22]. Electron microscopy (EM) image of MAP provided by Dr. Mike Collins and accessed at the www.johnes.org site.

MAP’s biological plasticity contributes directly to under-detection in human disease. While it exists as a classical acid-fast bacillus in ruminants, MAP in humans may adopt slow-growing or cell wall-deficient forms, including spheroplasts, which do not reliably stain or grow under conventional laboratory conditions [21,22]. This diagnostic limitation provides a plausible explanation for inconsistent detection and under-recognition in human disease Figure 1.

## 5. MAP Across the Neoplastic Continuum

**Figure 2:**
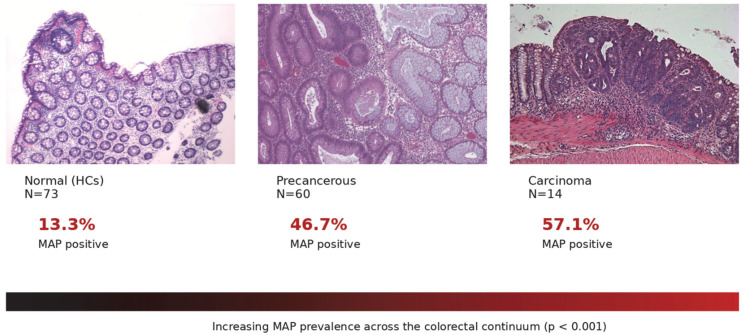
From Tehrani et al. [4]. Representative histologic sections illustrating the colorectal continuum from normal mucosa to precancerous lesions and invasive carcinoma, alongside corresponding rates of *Mycobacterium avium* subspecies *paratuberculosis* (MAP) detection. MAP positivity increases stepwise from 13.3% in healthy controls to 46.7% in precancerous tissue and 57.1% in carcinoma, demonstrating a significant upward trend (*p* < 0.001). These findings support an association between MAP presence and progressive colorectal neoplasia. Images curated from Wikipedia.

The study by Tehrani et al. represents the most direct evidence linking MAP to CRC [4]. Detection increases from controls to precancerous lesions to carcinoma, demonstrating a biologic gradient. The presence of MAP in adenomas and serrated lesions argues against passive colonization and is consistent with possible early involvement in tumorigenesis. Figure 2.

An earlier, underrecognized contribution to this field comes from the work of pathologist Ellen Pierce, who in 2018 postulated the presence of *Mycobacterium avium* subsp. *paratuberculosis* within colorectal tissues exhibiting dysplasia and carcinoma, based on histopathologic and molecular observations. Pierce proposed that MAP may function not merely as a bystander organism but as a persistent intracellular pathogen capable of driving chronic mucosal inflammation and epithelial injury, thereby contributing to neoplastic transformation [23]. Notably, Tehrani’s identification of MAP within structurally intact tissue compartments, rather than exclusively within necrotic or tumor-associated debris, supports a model of biologic participation rather than passive colonization. These observations, initially anticipatory and now corroborative, align with the detection gradient reported by Tehrani, strengthening the inference that MAP is detectable early in the neoplastic cascade rather than acting as a secondary opportunist.

## 6. Mechanistic Framework for MAP-Driven Carcinogenesis

MAP persists within macrophages and induces sustained inflammatory signaling, including activation of NF-κB and downstream cytokine cascades [24]. Chronic inflammation promotes epithelial proliferation, oxidative stress, and DNA damage, creating a permissive environment for neoplastic transformation. In parallel, MAP disrupts epithelial barrier integrity, facilitating immune activation and microbial translocation, further amplifying inflammatory signaling [11].

The relationship between inflammatory bowel disease (IBD) and CRC provides an established model of inflammation-driven carcinogenesis. CRC risk increases with disease duration and cumulative inflammatory burden, with historical estimates approaching 18% at 30 years of disease [25,26,27]. MAP’s association with Crohn’s disease provides a plausible indirect pathway via chronic inflammation and raises the possibility of a more direct contribution to carcinogenesis, although additional mechanistic and longitudinal studies are required.

An additional mechanistic dimension involves activation of human endogenous retroviruses (HERVs). HERV expression has been observed in colorectal cancer and is increasingly recognized as a contributor to genomic instability and oncogenic signaling [28,29]. MAP may activate HERVs through immune and epigenetic mechanisms, linking persistent infection to tumor-promoting pathways [30].

## 7. MAP in the Context of Infectious Carcinogenesis

**Figure 3:**
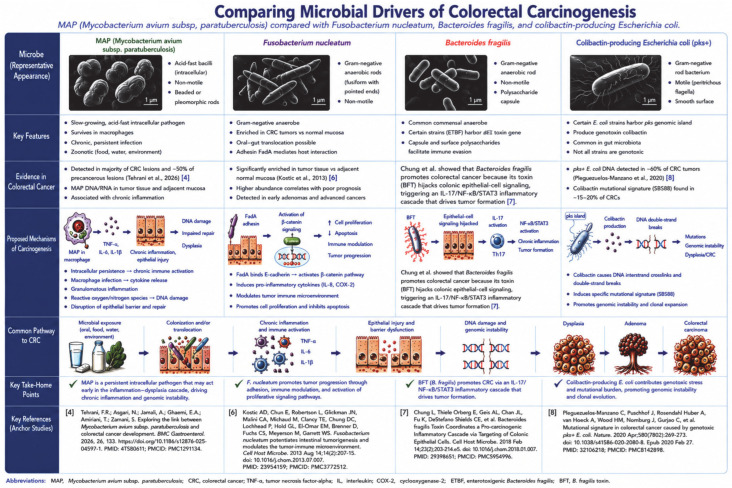
Microbial drivers of colorectal carcinogenesis.

MAP aligns with established microbial drivers of CRC, including *Fusobacterium nucleatum*, which modulates immune responses and β-catenin signaling, genotoxic *E. coli*, which induces DNA damage, and *Bacteroides fragilis*, which promotes inflammation [6,7,8]. Unlike these organisms, MAP exhibits systemic persistence and broad immunologic effects, positioning it as a potential upstream driver rather than a local cofactor (Figure 3).

This schematic positions *Mycobacterium avium* subsp. *paratuberculosis* (MAP) alongside established CRC-associated microbes: *Fusobacterium nucleatum* and colibactin-producing *Escherichia coli*, highlighting shared pathways of inflammation, epithelial injury and genomic instability. MAP is proposed as an early, persistent intracellular trigger that initiates the inflammation–dysplasia cascade, while *F. nucleatum* and pks^+^ *E. coli* primarily promote tumor progression and mutagenesis. Together, the figure supports a polymicrobial model of CRC in which distinct organisms contribute at different stages of carcinogenesis. Electron microscopy (EM) image of MAP provided by Dr. Mike Collins and accessed at the www.johnes.org site.

## 8. One Health Implications

MAP must be considered within a One Health framework; unlike many candidate oncogenic microbes restricted primarily to human transmission, MAP exists within a complex zoonotic ecosystem involving livestock, wildlife, agricultural runoff, water systems and food production. Large-scale surveillance studies demonstrate substantial MAP prevalence in dairy herds worldwide, resulting in persistent environmental shedding into soil and watershed systems. The organism’s resistance to environmental degradation and documented survival in water biofilms and some pasteurized dairy products raises the possibility of chronic low-level human exposure at the population scale [12,13,14].

MAP has also been associated with a range of chronic diseases, including Crohn’s disease, type 1 diabetes and multiple sclerosis [31,32]. These associations, spanning multiple organ systems, support the concept of MAP as a pleiotropic pathogen capable of influencing diverse disease processes, including CRC.

Within a One Health framework, CRC may therefore represent not solely an individual disease process, but potentially an additional downstream consequence of long-term ecological interactions among agricultural practices, food systems, microbial exposure and host susceptibility.

## 9. Experimental and Translational Opportunities

**Figure 4:**
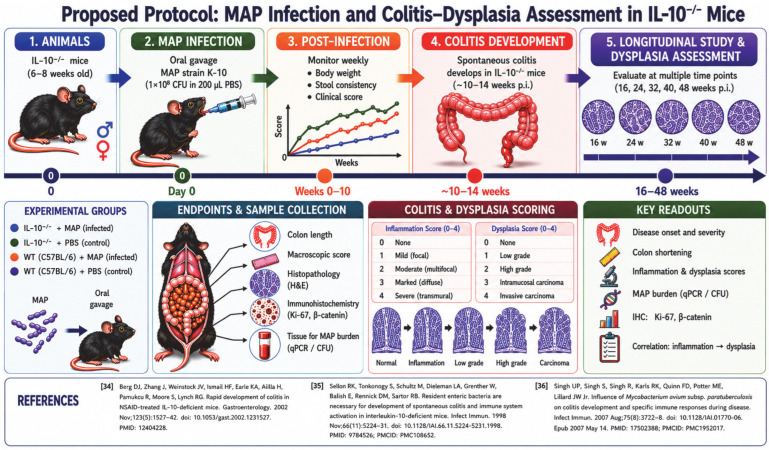
Proposed IL-10^−^/^−^ Mouse Model for MAP Infection, Colitis, and Dysplasia Assessment. Schematic overview of a proposed experimental protocol designed to evaluate the role of Johne’s disease-associated *Mycobacterium avium* subspecies *paratuberculosis* (MAP) infection in the development of chronic colitis and colorectal dysplasia using IL-10^−^/^−^ mice on a C57BL/6 background. Following weaning (3–4 weeks of age), mice undergo oral gavage with the MAP strain K-10 (typically 1–5 × 10^9^ CFU in 200 μL PBS). MAP infection may be confirmed by fecal qPCR and/or culture during early post-infection intervals. Beginning approximately 12 weeks after infection, colitis is induced and amplified using dextran sulfate sodium (DSS) administered in drinking water in cyclic exposures (e.g., 2.5% DSS, 7 days on/7 days off for three cycles). Animals are monitored longitudinally for body weight, stool consistency, occult blood, and clinical disease activity throughout the study period. At study completion (typically ≥36 weeks post-infection), mice are euthanized and tissues collected for downstream analyses, including histopathology, MAP burden assessment, immune profiling, and dysplasia scoring. Colonic tissues are evaluated for inflammatory severity, ulceration, architectural distortion, and neoplastic progression ranging from low-grade dysplasia to adenocarcinoma. Representative downstream analyses may include hematoxylin and eosin (H&E) histology, immunohistochemistry for Ki-67 and β-catenin, quantitative PCR for MAP detection, cytokine profiling, flow cytometry, and microbiome sequencing. The model is intended to investigate whether chronic MAP infection synergizes with impaired mucosal immune regulation and recurrent epithelial injury to accelerate inflammation-associated colorectal carcinogenesis.

Experimental models provide immediate opportunities to test the MAP–CRC hypothesis. IL-10-deficient mice develop inflammation-associated enterocolitis and colorectal cancer and have been used to study microbial drivers of disease [33,34]. MAP infection in this model offers a plausible system for evaluating progression from chronic infection to neoplasia [35]; the MAP/CRC protocol is illustrated in Figure 4.

In addition, extensive repositories of formalin-fixed, paraffin-embedded (FFPE) tissues represent a valuable resource for retrospective detection of MAP in well-characterized precancerous and cancerous specimens. Complementary analysis of fresh tissues would allow direct comparison with molecular findings and strengthen validation across methodologies.

## 10. Limitations and Counterarguments

The MAP/Public Health field remains controversial for several reasons; as discussed, detection methodologies vary substantially across studies, including PCR, culture, serology and phage amplification assays, each with differing sensitivity and specificity. MAP’s environmental ubiquity also raises concerns regarding laboratory contamination and false-positive molecular detection. Furthermore, some investigations have failed to detect MAP consistently in Crohn’s disease or other proposed MAP-associated conditions, contributing to continued debate regarding its pathogenic significance in humans.

An important alternative explanation of the Tehrani study is that evolving neoplastic tissue may create a permissive microenvironment favoring persistence or enrichment of MAP rather than MAP initiating disease. Altered mucosal barrier integrity, immune dysregulation and metabolic shifts within adenomas and carcinomas could theoretically enhance colonization by environmental organisms.

However, detection of MAP in precancerous lesions, the presence of a biologic gradient and mechanistic plausibility collectively argue against a purely epiphenomenal role. An additional limitation is the singular postulate and the single study directly evaluating MAP in colorectal neoplasia, with the available data derived from a limited geographic region. Broader multinational investigations using standardized detection methodologies will be necessary to determine reproducibility across populations. Accordingly, the present Perspective should be viewed as hypothesis-generating rather than confirmatory.

## 11. Conclusions

Controversy surrounding *Mycobacterium avium* subsp. *paratuberculosis* (MAP) in human disease has persisted for more than a century [36]. Although several studies have reported favorable responses to anti-MAP therapy in Crohn’s disease [37,38,39], the broader literature remains mixed, and MAP’s role in human pathology continues to be hotly debated.

The findings of Tehrani et al. provide an important new framework for reconsidering MAP in colorectal carcinogenesis. The demonstration of MAP across the colorectal neoplastic continuum, including precancerous lesions, strengthens the biologic plausibility of MAP as a potential infectious contributor to CRC rather than merely a late secondary colonizer. When viewed alongside established mechanisms of infectious carcinogenesis, chronic inflammation, microbial-driven genomic stress and emerging One Health concepts, these observations warrant serious further investigation.

At present, available evidence supports association rather than causation. MAP should therefore be regarded as a candidate infectious cofactor in CRC, with its precise pathogenic role remaining to be defined through mechanistic, longitudinal and multinational studies using standardized detection methodologies. Importantly, proposed investigations using the preclinical IL-10^−^/^−^ model may help clarify whether MAP directly contributes to inflammation-driven tumorigenesis and provide mechanistic insight into its potential role in colorectal cancer.

Should future studies confirm a contributory role for MAP in colorectal carcinogenesis, the implications would extend beyond CRC itself, reshaping understanding of microbially mediated cancer and opening new avenues for prevention, early detection and targeted intervention. In that sense, colorectal cancer may indeed help put MAP on the map.

## Data Availability

No new data were created or analyzed in this study.
